# Prolongation of Anesthesia with Hyper-Volatile Anesthetics

**Published:** 1910-06-15

**Authors:** Carl G. Parsons

**Affiliations:** Denver


### PROLONGATION OF ANESTHESIA WITH HYPERVOLATILE ANESTHETICS.

BY CARL G. PARSONS, M.D., DENVER.

When giving a single administration of hypervolatile or short-term anesthetics, such as ethyl chlorid, nitrous oxid, or somnoform, etc., the great difficulty confronting the anesthetist and operator is to keep the patient in the third or surgical anesthetic stage a sufficient length of time to enable the operator to complete certain comparatively short operations about the mouth and upper air passages, such as extracting a number of teeth, tonsil dissections, adenectomies, etc. The average available anesthesia under nitrous oxid is about 30 seconds; with ethyl chlorid about 1 minute; with somnoform, 60 to 100 seconds. Of these three main so-called short-term anaesthetics this paper deals *particularly* with somnoform, since it is the most potent and lasting; for besides being composed of a base ethyl chlorid (60 per cent.) and methyl chlorid (35 per cent), there is also contained in the mixture 5 per cent. of ethyl bromid, a drug which is likened to chloroform in that it possesses powerful sedative and analgestic properties. Ethyl bromid is *the* one drug which produces the analgesic state following the anesthetic proper.

Any one of these hypervolatile anesthetics may be used in producing prolonged anesthesia for from 15 minutes to 1 or 2 hours, where operations are performed upon parts of the body *other* than the mouth and upper air passages. However, after about six years of study and experience in anesthetics and their administration exclusively, I have found the following methods to be satisfactory for producing *prolonged* anesthesia in oral and throat surgery.

*First.*—Administering the drug with due regard to the law of anesthetic accommodation.

*Second.*——Changing the size of the inhaling apparatus to conform with the age of the patient.

*Third.*—Varying the dosage.

*Fourth.*—-Posture.

*Fifth.*—By using the vapor from a bag connected by tubing to a Hewitt's mouth-gag, dental prop, or metal mouth tube.

First.—Gradual continuous administration of *any* anesthetic, with equal drug distribution, is one of the most important principles in anesthetization. A safer anesthesia is maintained, and at the same time the patient can be more thoroughly and safely charged with the drug, thus insuring a longer available anesthesia. The administrator better knows his patient's narcotic susceptibility; the anesthesia is more uniform; and there are no extreme fluctuations in the percentage of vapor inhaled, but a gradual scientific anesthetic state brought about, which insures a deeper and more lasting narcosis.

Second.—The ordinary somnoform inhaler is built for adults. If an apparatus of the same size is used for young children, the intake of vapor from the bag is not sufficient to properly anesthetize them, owing to their breathing amplitude being so much smaller than that of an adult. A smaller bag should be used (and smaller face-piece), thus enabling them to get their proper dosage.

Third.—The dosage should be varied. The 3 c.c. glass capsules of somnoform are not of sufficient size for any prolonged anesthesia. It is immaterial how much vapor is in the bag, whether it be 5 c.c. or 20 c.c., only as a matter of economy; the anesthesia is determined solely by the physiological signs exhibited by the patient during inhalation. Therefore, the dosage should be measured to suit the build of the patient and inhalation should take place gradually until signs of anesthesia are well marked.

Fourth.—Patients in Trendelenburg's posture (patient resting on a 45 degree inclined table with legs and feet hanging over the highest end of table) will hold longer under a single administration of a hypervolatile anesthetic than when in the sitting position. The hydrostatic effect of gravity keeps the vapor in the cerebral circulation longer when the head is low. It is true that the volume of blood in the brain is not altered, but the velocity of the flow is retarded. A partial cerebral CO2 narcosis is present in conjunction with the anesthesia.

Fifth.—When a continuous administration of somnoform is desired for from five to ten minutes, as, for instance, when a tonsil dissection is done, a great number of teeth need extracting, oral and throat operations, etc., the vapor is conducted from a rubber bag to a special mouth-gag, prop, or metal tube, by tubing. As the patient inspires, the vapor is pumped into the mouth, a sufficient quantity being given to cause the proper physiological phenomena of the eyes to take place. When the patient is once well nnder, it is surprising to note the small quantity of drug uecessary to maintain a surgical degree of anesthesia. One point of particular importance with this method is to pump the vapor during the *entire* period of the patient's inspirations.

Maintaining anesthesia for nose, mouth, and throat surgery is very difficult; therefore, the operator should be on the alert and ready to commence with the operation as soon as the anesthetist removes the mask. When dealing with hypervolatile anesthetics, seconds are minutes and minutes are hours.—*Items of Intererst,*—Dec. 1909.

